# Infantile myofibromatosis treated by mandibulectomy and staged reconstruction with submental flap and free fibula flap: a case report

**DOI:** 10.1186/s40463-019-0333-z

**Published:** 2019-03-14

**Authors:** Alexandra Maby, Benoit Guay, François Thuot

**Affiliations:** 1 Département d’ophtalmologie et d’oto-rhino-laryngologie – chirurgie cervico-faciale, Faculté de Médecine, Université Laval, 1050, avenue de la Médecine, Québec, QC G1V 0A6 Canada; 2 CHU de Québec, Hôpital L’Hôtel-Dieu de Québec, 11 Côte du Palais, Québec, QC G1R 2J6 Canada

**Keywords:** Infantile myofibromatosis, Myofibroma, Mandibular reconstruction, Submental island flap, Fibula free flap

## Abstract

**Background:**

Infantile myofibromatosis is the most common benign fibrous tumor in infants. Three different types have been reported in the literature. The most commonly affected areas are the head, the neck and the trunk. Our patient showed a very high level of mandibular destruction resistant to all mandibular sparing treatment strategies requiring segmental mandibulectomy and complex reconstruction.

**Case presentation:**

We describe a rare case of multicentric infantile myofibromatosis with mandibular bone destruction. The treatment required a succession of chemotherapy, a subtotal transoral resection and a hemi-mandibulectomy. The mandibular reconstruction was staged with initial bridging titanium plate with a submental flap, followed later by a fibula free flap.

**Conclusion:**

Mandibular involvement by myofibromatosis is rare, and the extend of bone destruction and reconstruction make this case unique. To our knowledge, this is the only reported case of fibula free flap mandibular reconstruction in a patient with infantile myofibromatosis , as well as one of the youngest reported submental island flaps for any pathology. We describe the clinical presentation and management, including relevant imaging, histopathology, medical and surgical treatment as well as a review of relevant literature.

## Introduction

Infantile myofibromatosis (IM) is characterized by benign tumoral proliferations of fibroblasts and/or myofibroblasts. IM was first described in 1954 by Stout as a “generalized congenital fibromatosis” [1]. In 1981, Chung and Enzinger discovered its myofibroblastic origin and used the term infantile myofibromatosis for the first time [2]. Although IM is rare, it is the most common fibrous tumor in the first year of life [3]. IM usually presents as firm, purple or flesh-colored nodules in the skin and subcutaneous tissue. The most commonly affected body areas are the head, the neck, and the trunk [4, 5]. The exact etiology of the condition is unknown, and most cases reported are sporadic. We present a unique case of IM with mandibular destruction which appears unique for 2 reasons. First, although this pathology is benign and frequently indolent, our patient showed a very high level of mandibular destruction resistant to all mandibular sparing treatment strategies. Second, his very young age at presentation (6 months) and resection (18 months) made reconstruction highly challenging.

## Case report

### Case presentation

**Fig. 1:**
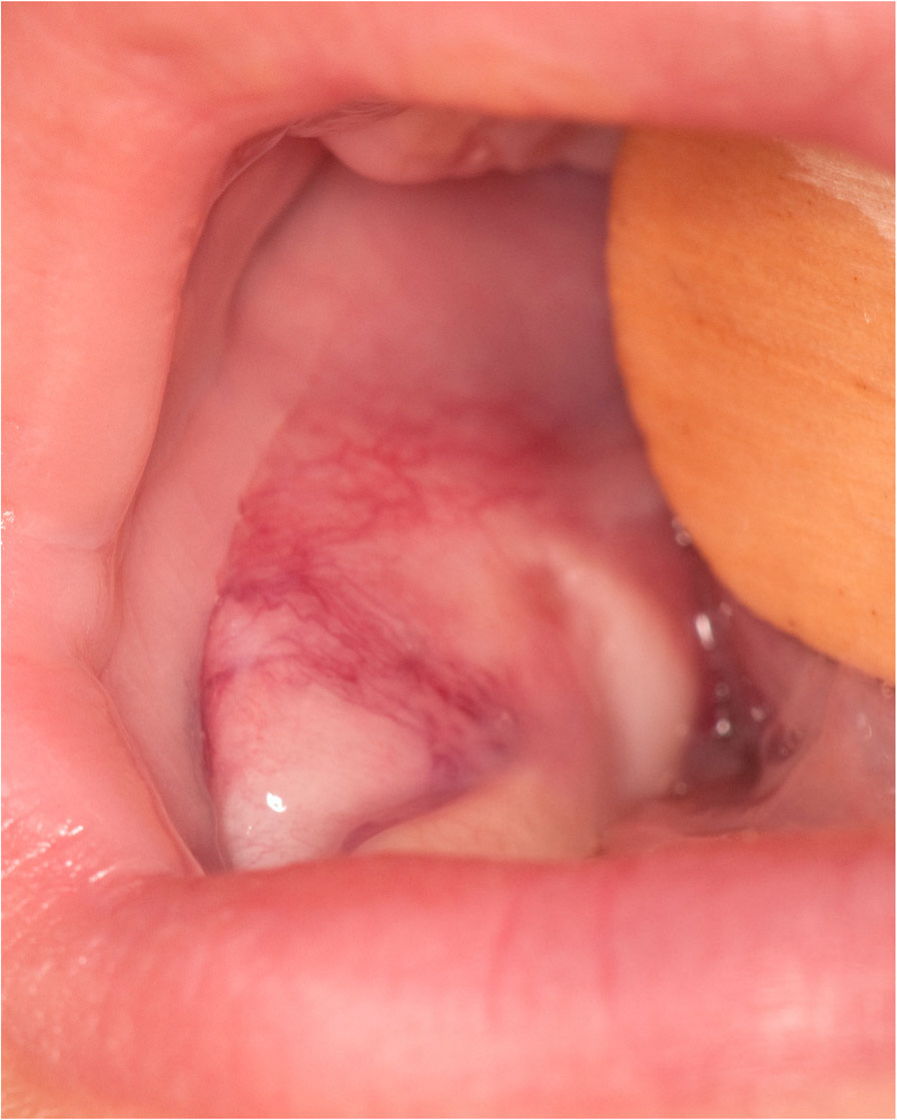
Soft tissue submucosal mass of the right mandible

A 6-month-old boy was referred for a right lower cheek mass and a left thoracic subcutaneous mass both present for 3 months. He was asymptomatic and healthy with no significant medical, surgical or familial history. The thoracic lesion was small (1,0 × 1,6 cm) and mobile. The cheek lesion presented as a deep and firm soft tissue submucosal mass adherent to the mandible (Fig. 1).

### Radiological findings

**Fig. 2:**
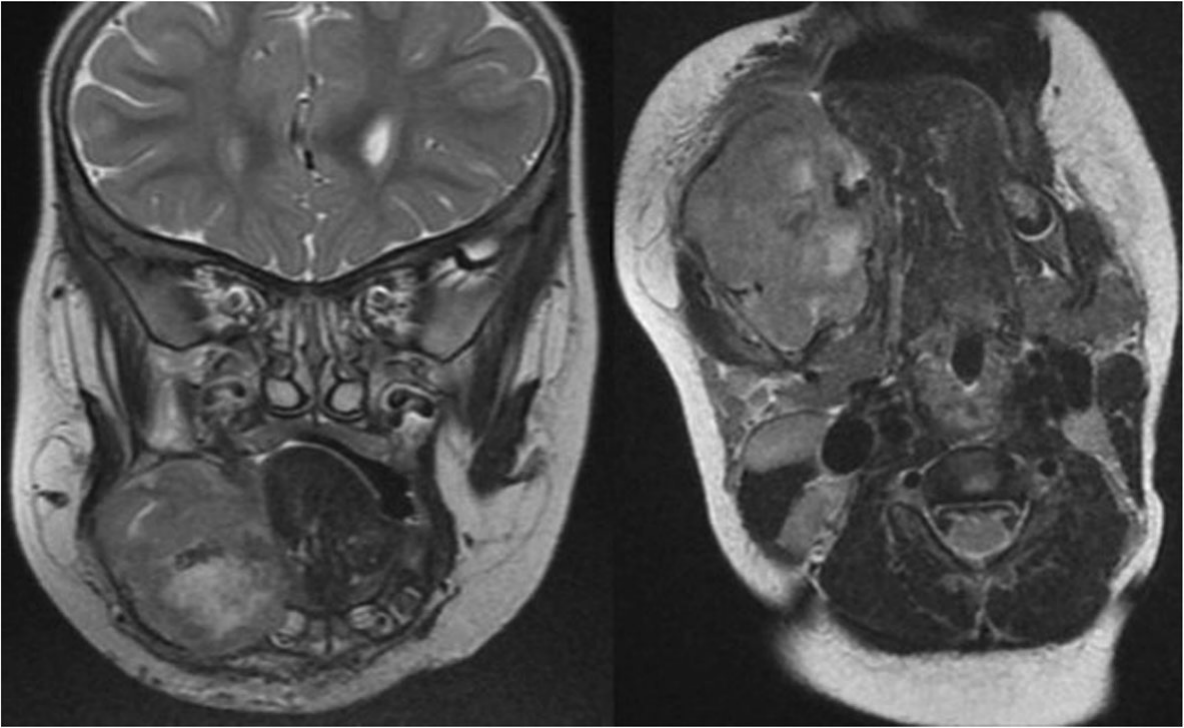
MRI showing a soft tissue mass with necrotic center located in right buccinator and masseter muscles and bony invasion of the right mandible

Ultrasound (US) examination of both masses showed hypoechoic lesions with small calcifications and scant vascularization. Magnetic resonance imaging (MRI) of the neck showed a soft tissue mass of 3.4 (AP) × 2.2 (T) × 3.8 cm (CC), with lobulated contours and a cystic center (Fig. 2). The lesion was located within the right buccinator and masseter muscles and showed bony invasion of the right mandible and peripheral enhancement with injection of gadolinium. To rule out other synchronous lesions, cardiac and abdominal US and brain MRI were performed and were negative.

### Histopathology

**Fig. 3:**
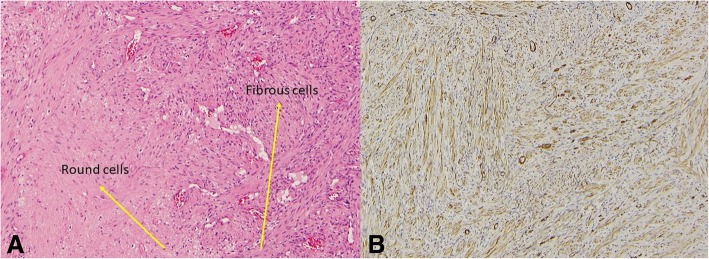
**a**: Mixture of spindle (fibrous) cells and round (histiocytic) cells arranged in a storiform pattern. **b**: Immunostaining: positive for smooth muscle actin and Hhf35

The thoracic lesion was excised and the oral lesion biopsied. The histopathology was similar and showed spindle cell tumors with a storiform pattern (Fig. 3) compatible with infantile myofibroma.

### Management

**Fig. 4:**
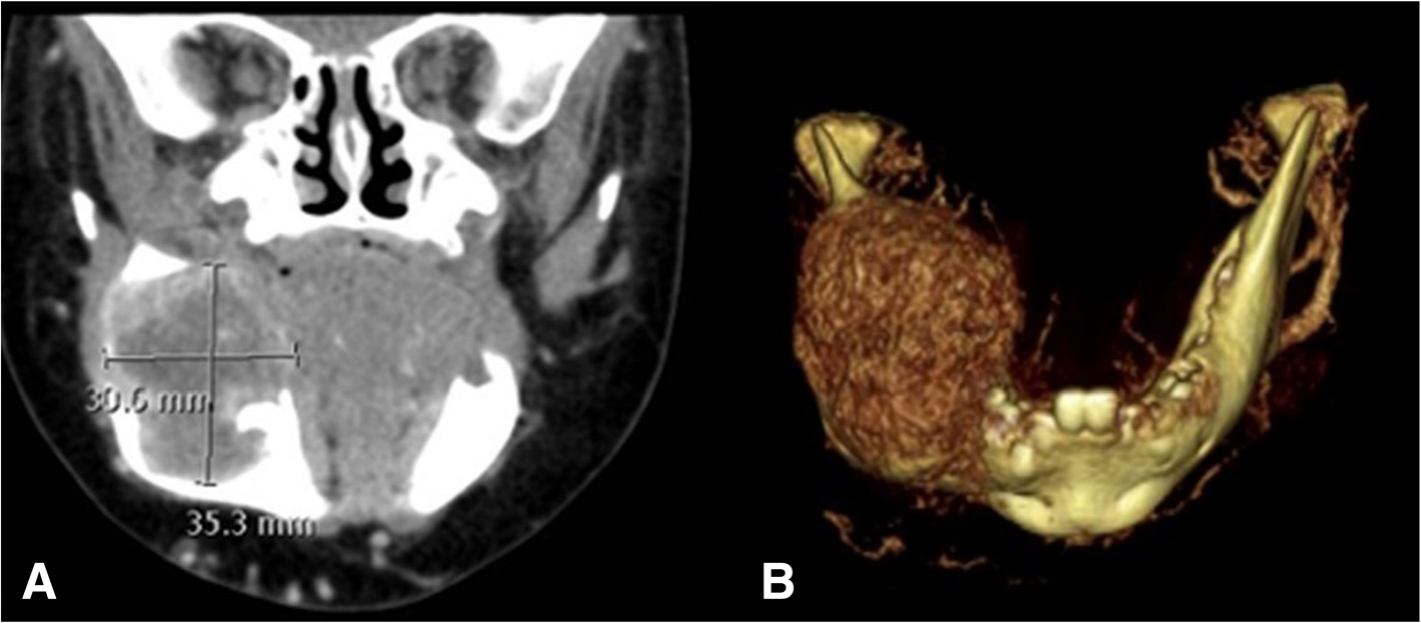
**a**: CT scan shows progression of the lesion with extension to the intermaxillary commissure and deep mandibular erosion. **b**: 3D reconstruction

Throughout the treatment, the conduct was coordinated by an adult head and neck oncology – reconstructive otolaryngologist and a pediatric oncologist. On multiple occasions, the patient was presented at a multidisciplinary pediatric oncology clinic for medical aspects and at a multidisciplinary head and neck oncology clinic for surgical aspects. The first surgery (debulking) was done jointly by an adult head and neck oncology - reconstructive otolaryngologist and a pediatric otolaryngologist. All other ablative and reconstructive surgeries were performed jointly by two adult head and neck oncology - reconstructive otolaryngologists. Surgical treatment of the mandibular tumor was initially judged too morbid and chemotherapy was started with Methotrexate and Vinblastine. After six cycles, the patient presented feeding difficulties. A computerized tomography (CT) Scan was performed at this time and showed a progression of the lesion with extension to the retromolar trigone and deep mandibular erosion (Fig. 4).

**Fig. 5:**
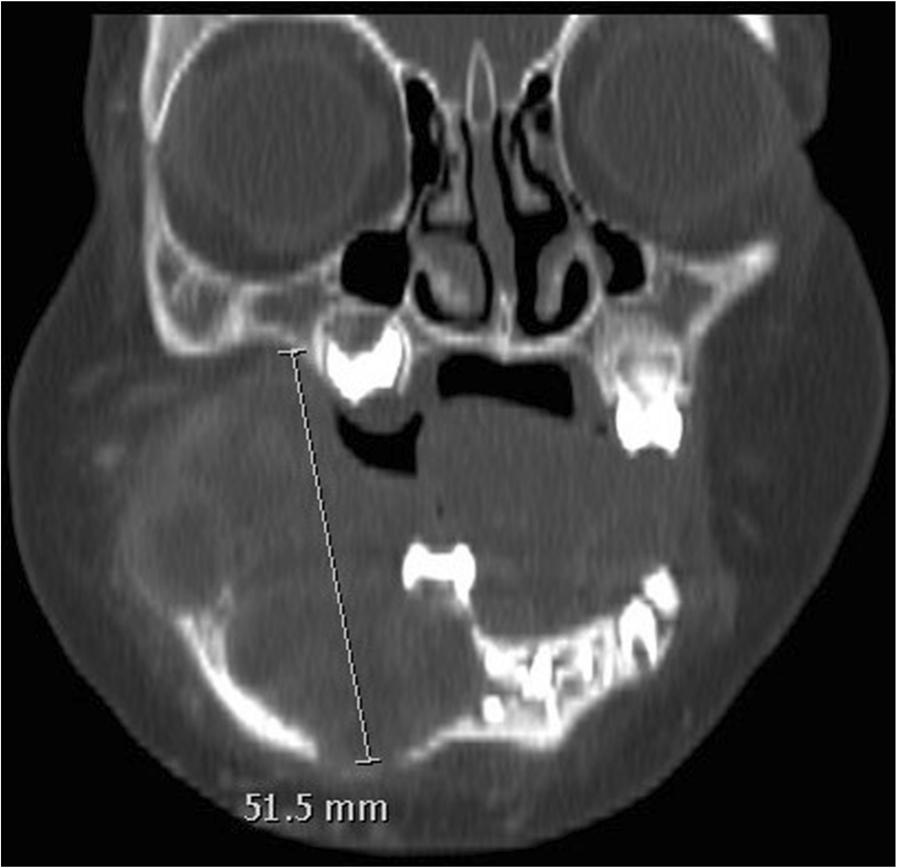
CT scan shows progression of the tumor with new extension along the right maxilla despite oral excision and chemotherapy (coronal view)

Chemotherapy was suspended, and a conservative trans-oral resection of the tumor was done. Only the intraoral exophytic portion of the tumor was excised to allow jaw closure and occlusion on the contralateral side. No other structures were resected, and the main specimen size was 3.5 X 2.8 X 2.5 cm. The surgery was well tolerated with no complications. One month later, an MRI showed progression of the tumor reaching 4.0 (AP) × 3.2 (T) × 4.1 (CC) cm with extension to the medial pterygoid muscle and infiltration of the alveolar nerve. Chemotherapy with Methotrexate and Vinblastine was pursued. Four months later, despite the chemotherapy, the patient had weight loss because of recurrence and progression of the intraoral mass affecting the oral phase of swallowing as well as preventing contralateral occlusion contact. The control MRI demonstrated progression of the tumor now reaching 5.3 (AP) × 3.9 (T) × 4.9 (CC) cm with new tumor extension along the right maxilla and an increased recruitment of peripheral vasculature (Fig. 5).

**Fig. 6:**
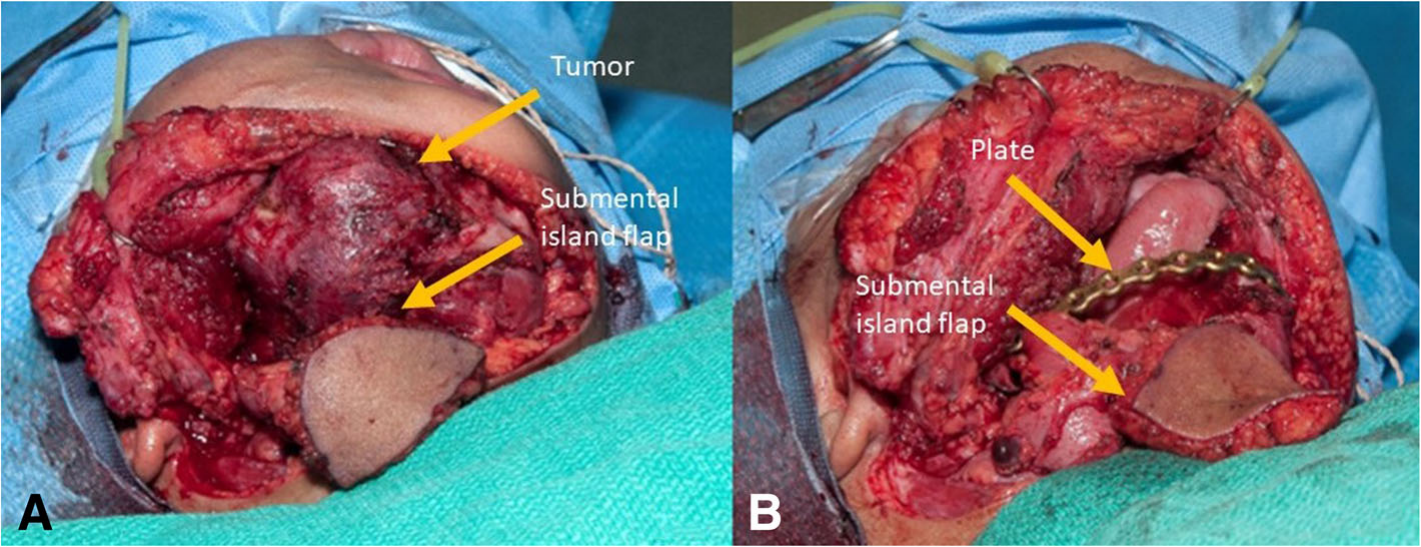
**a**: Lesion before resection. **b**: Post resection of the tumor showing mandibular reconstruction with a titanium plate

Faced with tumor progression refractory to chemotherapy and conservative surgery, radical excision with segmental mandibulectomy was planned. We decided to stage the reconstruction because of the patient’s age (18 months) and the local aggressiveness of the disease. It was resected with conservative margins and we planned the final reconstruction later when local control was achieved. Also, there are very few precedents of mandibular free flap before 2 years, and the impact on mandibular development at this early age is not well documented. The risk of valgus ankle deformity is significant before 8 years and decreases with age. It can be prevented or corrected by a synostosis. This was considered optional by pediatric orthopedics, only if late deformity would occur. Delaying the free flap to 42 months was therefore judged a good compromise, minimizing the risk of weight-baring plate complications on a solid diet. A combined trans-oral trans-cervical segmental mandibulectomy was done with preservation of the condyle (Fig. 6). A temporary mandibular reconstruction was achieved with a bridging titanium plate for the bony defect and intraoral reconstruction with a submental island flap. The plate was adapted to the outer mandibular cortex before osteotomies without preoperative 3D planning. There were no complications and the evolution and function were excellent until definitive bony reconstruction.

**Fig. 7:**
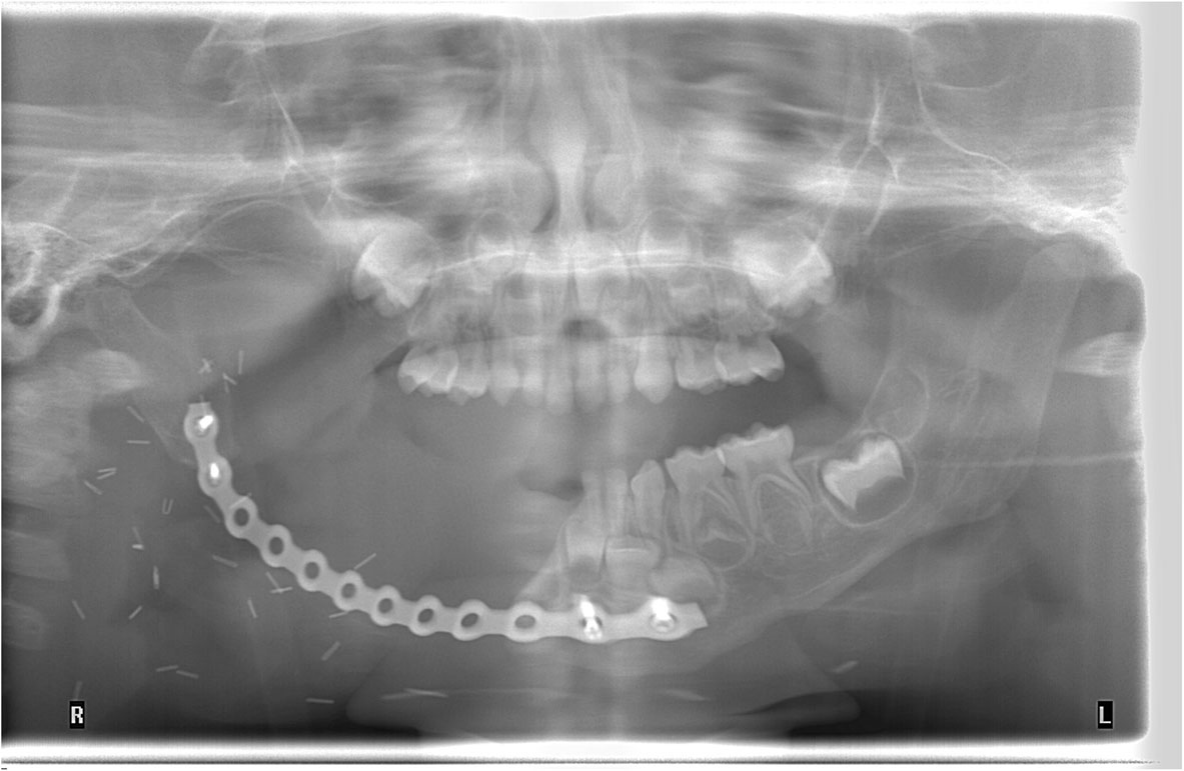
Post-operative panoramic radiograph after ablation

Pathology confirmed a 5.6 (CC) × 4.8 (AP) × 3.6 (T) cm myofibroma with gross mandibular invasion. Margins were close but negative. Despite the invasive nature of the tumor, there was no evidence of cancer. The immunostaining was positive for smooth muscle actin (strong and diffuse) and Hhf35 (moderate to strong and local). Rare cells were positive for desmin while the markers caldesmon, MYOD-1, myogenin, CD34 and AE1-AE3 were negative. Subsequently, a control MRI showed no recurrence of the lesion. Because the mandibulectomy spared the condylar growth center, vertical and horizontal remodeling occurred within this region and no drift occurred between 18 and 42 months. The patient retained a functional occlusion on the left side (Fig. 7).

**Fig. 8:**
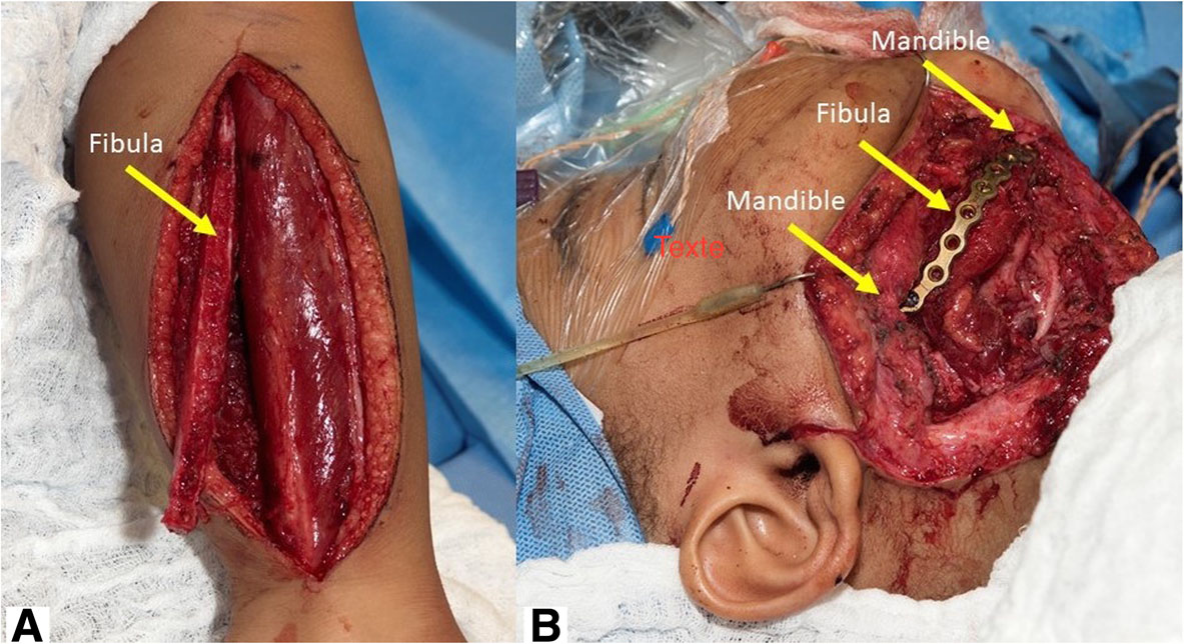
**a**: Fibula free flap harvest. **b**: Flap inset, fibula fixed to the mandible with the same titanium plate

**Fig. 9:**
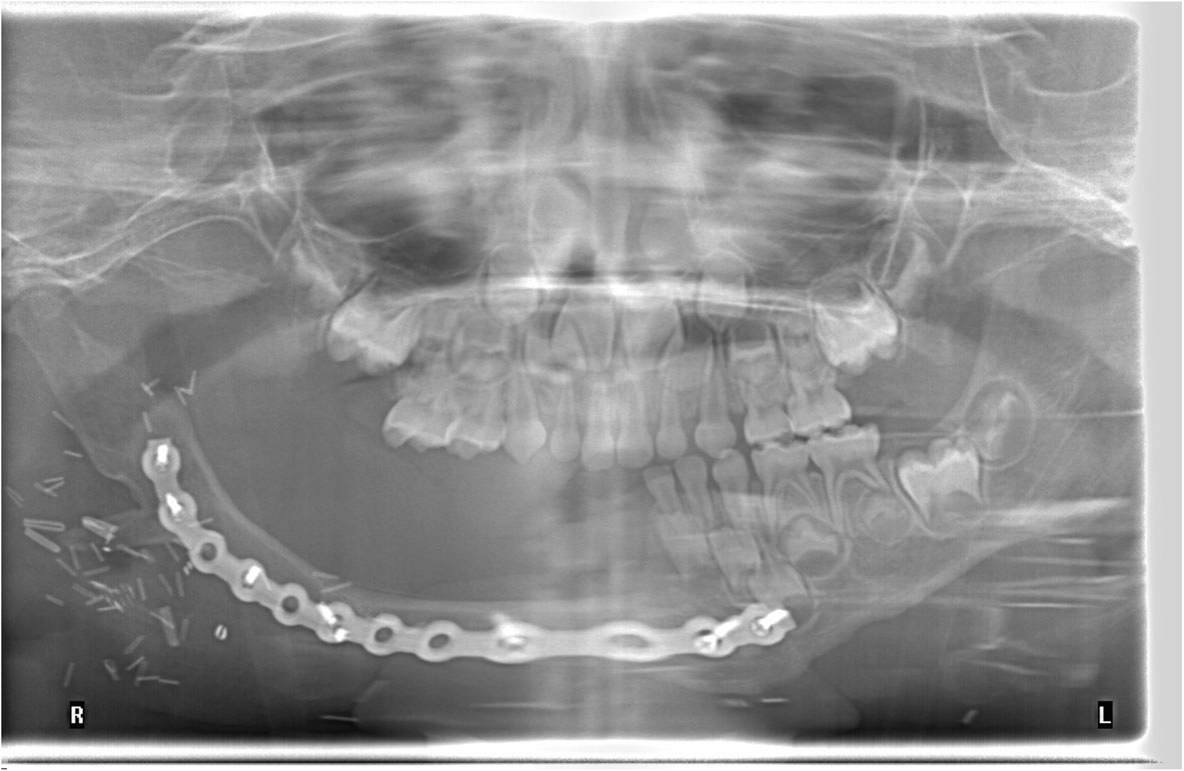
Postoperative panoramic radiograph after fibular free flap reconstruction

**Fig. 10:**
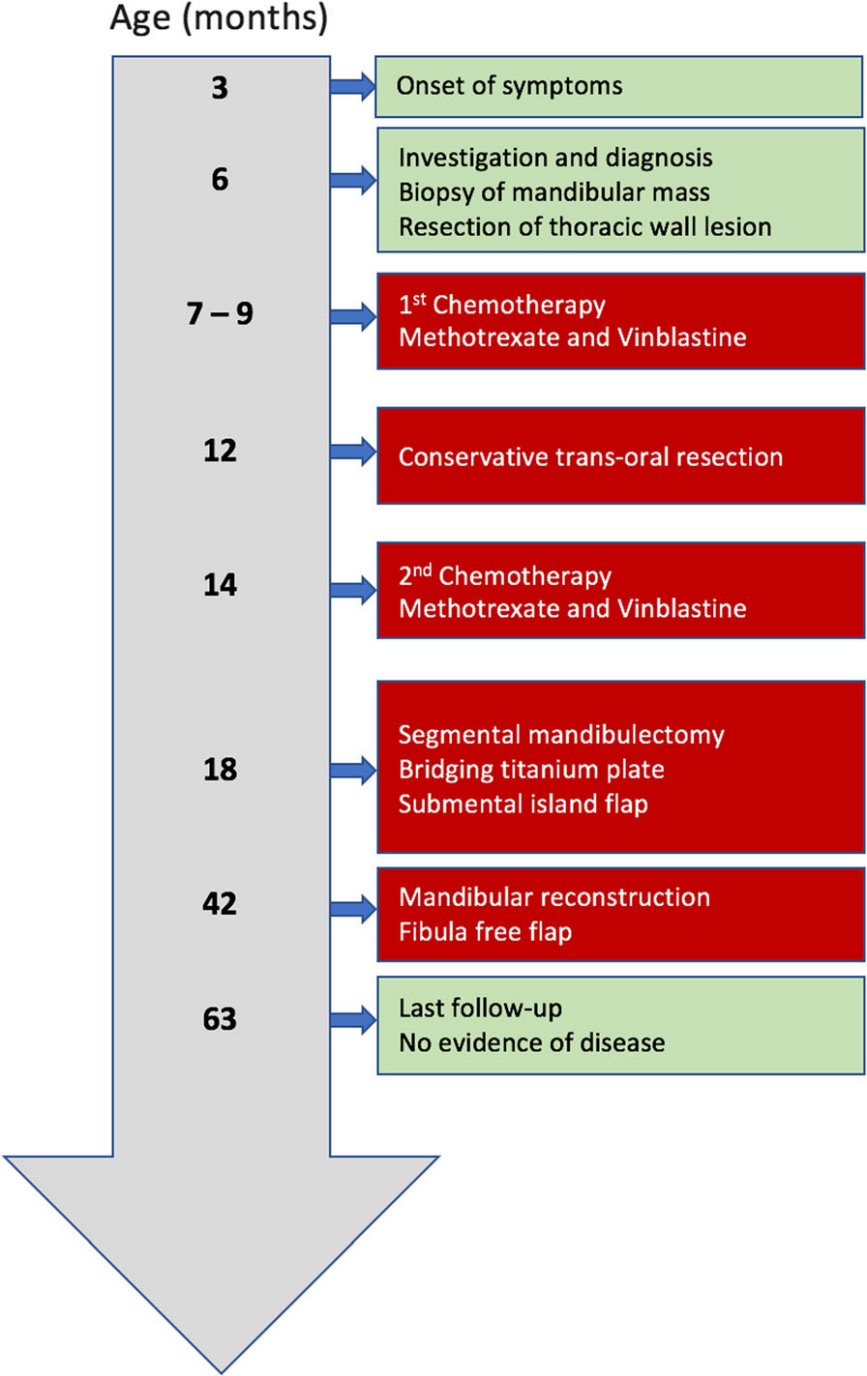
Timeline of evolution and treatments

At 42-month-old, a delayed microvascular bony reconstruction with a fibula free flap was completed, surgical exploration confirming remission of the tumor (Fig. 8). At the time of the reconstruction with the fibula free flap, the exploration revealed a well-tolerated and stable titanium plate with new bone formation around it at both osteotomy sites. To facilitate the procedure and protect the occlusion, it was left in place and used to fix the fibula to the defect. Later removal was planned. The patient had a favorable postoperative evolution. He resumed oral diet three days post op and was discharged from the hospital eight days postop. The only complication was a minor skin dehiscence from the fibula donor site, which completely healed treated with water gel and wound care. He later had 2 revision surgeries without complications, one for titanium plate removal and one for skin paddle thinning. At the time of plate removal, a complete and solid bony union was found at both osteotomy sites well as new bone formation along the reconstruction plate (Fig. 9). At the last follow-up at 5^3/12^ years-old and 4 years after the resection, he was doing well and free of disease with an excellent function. Figure 10 is a timeline that summarizes the patient’s evolution and treatments.

## Discussion

A scoping review was performed in Medline/Pubmed database. English and French articles were searched using the keywords “myofibromatosis” “myofibroma” “infantile” “head and neck” and “mandible” for the pathology and “mandibular reconstruction” “pediatric” “free fibula flap” and “submental island flap” for the reconstruction. The snowballing method was also applied to selected articles. Despite being a rare pathology, IM is the most common fibrous tumor of infancy [3]. In the past, this disease has also been called congenital fibrosarcoma [6], congenital generalized fibromatosis, generalized harmartomatosis, multiple congenital mesenchymal tumors, diffuse congenital fibromatosis, and multiple vascular leiomyomas of the newborn [2]. Approximately 300 cases of IM have been reported in the English literature [5]. 30 to 50% of cases are diagnosed at birth or during the neonatal period [2, 7, 8]. IM is characterized by the formation of nodules or masses in skin, subcutaneous tissue, muscle, bone and viscera (mainly gastrointestinal, pulmonary, and cardiac) [9–11]. According to Chung, the main anatomical sites affected are the head and neck region (33%), the trunk (33%) and the limbs (31%) [2].

Three different clinical forms of IM have been defined: solitary and multicentric, with or without visceral involvement. Solitary IM is characterized by a single nodule and is the most frequent presentation [2]. Multicentric IM without visceral lesions involves several nodules in the skin, subcutaneous tissues, muscles and bones. The prognosis of these two forms is generally excellent with conservative surgery and can show spontaneous regression [12]. IM with visceral involvement represents 15–20% cases and is defined by visceral lesions in addition to skin nodules. The prognosis is associated with high morbidity and mortality despite surgery and chemotherapy [2]. Rapidly growing tumor cause visceral compression leading to gastrointestinal and cardiopulmonary compromise, whereas perivascular nodules interfere with organs blood supply [13, 14]. Familiarity with the recognition of the three clinical forms is important, requiring different management strategies. Most of these tumors are sporadic and isolated. Rare familial cases of IM have been described and mutations of 2 genes (PDGFRB and NOTCH3) have been identified causing the disease [15–17].

Diagnosis can be suspected based on family history and physical examination but is made chiefly by biopsy [2]. Histopathology examination reveals interlacing fascicles of spindle cells (myofibroblasts) in the periphery, forming nodules separated by collagen tissue with no nuclear atypia [3, 18]. Characteristics on imaging include a mass with an anechoic center on ultrasound, low signal on T1-weighted imaging and high or low signal intensity areas on T2-weighted imaging on MRI and a mass with peripheral enhancements and calcifications in contrast enhanced CT scan [19].

Due to the benign nature of IM, therapies producing the least long-term sequels and toxicity are preferred. Conservative surgery is the treatment of choice for the solitary form when morbidity and complications are minimal. In cases of incomplete resection, re-excision can be proposed later [20]. Treatment for multicentric IM is not well defined. For lesions affecting the skin and/or muscles only, a wait-and-see policy is often proposed because of a tendency towards spontaneous regression [21]. Radical surgical excision is required if the lesions are symptomatic or potentially life-threatening.

Chemotherapy is considered for solitary lesions when surgery is judged too morbid or for multicentric progressive disease. Standard regimen is a combination of methotrexate and vinblastine [1, 22]. As summarized by Levine et all [12], several reports describe response and long-term success with this protocol, initially used to treat desmoid tumor allowing regression or stabilization of the lesions with no severe toxicity. This regimen is often chosen as no late effects have been described with these drugs. No large or multicentric series are available. Other treatments such as IFN-alpha or conventional chemotherapy (vincristine, actinomycin D, and cyclophosphamide) should be considered only for disease refractory to standard protocols or with rapid progression because of the long-term risks of secondary malignancy [14, 22].

Myofibroma of the oral cavity occurs mainly in the mandible (38%) and less frequently in the lips, cheeks and tongue [7]. It is typically diagnosed in children in the first decade of life (mean 7.2 years) with a male predominance (male/female ratio 2.1:1) [23]. These features vary from those found in myofibromas of the oral mucosa, which is diagnosed in an older age group (mean 21.7 years) with a female predominance (female/male ratio 1,6:1) [24].

Symptomatology depends on the tumor location. Patients with myofibroma of the mandible usually present with asymptomatic jaw swelling, which is occasionally accompanied by an intra-oral soft tissue mass. The initial growth can be aggressive in 24% of cases [8]. Radiological findings are non-specific but are useful to delineate the extension and the progression of the tumor.

Thirty-three cases of myofibroma of the mandible have been reported in literature (Table 1) [4, 23–43] with twenty-eight cases of infantile myofibromatosis and five cases of adult myofibromatosis. Seven cases had missing data. Twenty-two patients were treated with complete primary local resection without reconstruction. Three cases of mandibular reconstruction with iliac crest bone grafting were reported in two adults and one child [30]. No recurrence during the follow-up period (6 months to 17 years) was observed in any patient.

**Table 1:** Review of Literature of infantile myofibromatosis of the mandible

Authors (year)	N	Age	Male/Female	Treatment	Reconstruction	Evolution
Slootweg P and al. (1984) [25]	1	Newborn	Male	Complete surgical excision	None	Free of disease for 10 years
Maj Mark S and al. (1990)	1	6y	Male	Complete surgical excision	None	Free of disease for 28 months
Inwards and al. (1991) [34]	3	6 mo-16y	–	Complete surgical excision	None	Free of disease for 2–5 years
		6 mo-16y	–	Complete surgical excision	None	
		6 mo-16y	–	Complete surgical excision	None	
Nadarajah Vigneswaran and al. (1992)	3	2y	Female	Complete surgical excision	None	Free of disease for 6 months
		11y	Female	Complete surgical excision	None	Free of disease for 1 year
		6y	Male	Complete surgical excision	None	Free of disease for several years
Jones and al. (1994) [35]	3	5mo	Male	–	–	–
		8y	Male	–	–	–
		14y	Female	–	–	–
Lingen and al. (1995) [36]	1	–	Female	Complete surgical excision	None	Free of disease for 6 years
Sugatami and al. (1995) [37]	1	2mo	Male	Complete surgical excision	None	Free of disease for 3 years
Loundon N and al. (1999) [4]	1	9y	Male	Complete surgical excision	None	Free of disease for 30 months
Montgomery and al. (2000) [38]	2	1y	Male	–	–	Free of disease for 4 years
		9mo	Male	–	–	Free of disease for 15 months
Olivier and al. (2003)	1	34y	Female	Complete surgical resection and iliac crest block graft	Yes	–
Maria J. Troulis and al. (2004) [30]	1	6,5y	Male	Complete surgical resection Iliac crest block graft	Yes	–
Sedghisadeh and al. (2004)	1	20y	Male	Complete surgical resection and iliac crest block graft	Yes	–
Odell and al. (2004)	1	10y	Male	–	–	–
I. Chtourou and al. (2006)	1	11y	Female	Complete surgical excision	None	Free of disease for 3 years
I Allon and al. (2007) [24]	4	5mo	Female	Complete surgical excision	None	Free of disease for 1,5 years
		7y	Female	Complete surgical excision	None	Free of disease for 6 months
		4,5y	Male	Complete surgical excision	None	–
		4,5y	Male	Complete surgical excision	None	Free of disease for 17 years
S Ech-Charif and al. (2008) [33]	1	1,5y	–	–	–	–
Ramadorai and al. (2010)	1	32y	Female	Complete surgical resection and reconstruction with a titanium plate	Yes	–
Nouri and al. (2011) [32]	1	16y	Male	Complete surgical excision	None	–
Brierley and al. (2013) [42]	1	43y	Female	Complete surgical excision	None	–
Lee and al. (2014) [43]	1	31y	Female	Complete surgical excision	None	–
V. Venkatesh and al. (2015) [27]	1	11y	Male	Complete surgical excision	None	–
R Lopez and al. (2015)	1	2y	Female	Complete surgical excision	None	Free of disease for 4 years
H Castro and al. (2016) [31]	1	13y	Female	Complete surgical excision	None	–

Before the advent of bony free flaps and rigid reconstruction plates, children with benign or malignant jaw tumors were preferentially reconstructed by the placement of a bone graft and immobilization with maxillomandibular fixation [44, 45]. Although this strategy provided a favorable and functional result in a single operation, it was also associated with high infection rates and insufficient bone stock for dental rehabilitation [46, 47]. The use of rigid reconstruction plates allows a negative margin resection while preserving the occlusion until a definitive bony reconstruction is planned in the absence of tumor recurrence [30]. For the present case, it also allowed growth to an age permitting the success of a free flap. Since the intraoral defect was extensive, a submental island flap was done at the time of the mandibulectomy for soft tissue reconstruction.

The strategy of staging mandibular reconstruction using a plate and submental island flap followed by a delayed fibula free flap is novel. To our knowledge, this is the only reported case of fibula free flap mandibular reconstruction in a patient with IM, as well as one of the youngest reported submental island flaps for any pathology. The later was first described by Martin et al. in 1993 [48]. In a review in 2014, Rahpeyma et al. described several variants based on 90 published studies [49]. Its use is rare in the pediatric population, the youngest being at the age of 6 weeks for closure of a skull base defect from resection of a teratoma [50, 51]. In 1993, Posnick et al. reported the first free fibula flap in the reconstruction of pediatric mandibular defect [52]. Since then, cases in patients as young as 10-month-old have been reported [53]. Stelnicki et al. even reported the bilateral mandibular reconstruction with two fibula free flap in a 2 ½ year-old patient with severe craniofacial malformation [54]. Several papers testify the reliability of free fibula flap in children, with more than 50 patients reported under the age of 18 years old [55–60].

According to an anthropological study, mandibular width and height increases rapidly before 4 years of age and between 8 and 12 years [61]. The growth potential of the reconstructed mandible is driven from the residual mandible and the condyle remains the most reliable growth center [61–65]. Good functional and aesthetic outcomes following mandibular reconstruction with vascularized fibula flap have been reported by Crosby and al [64]. If the native mandibular growth plate is preserved, the transferred fibula will accommodate itself as the child grows without interfering with the growth pattern of the lower and mid face. Preservation of the condylar epiphyseal plate should be a priority before its fusion at the age of 18 to maximize proper craniofacial development. Although experimental concerns of lower limb growth discrepancies have been raised from pediatric fibula harvesting, there is no clinical demonstration of such a phenomenon in the literature. There is a significant risk of valgus ankle deformity before 8 years, which can be prevented or corrected by performing an immediate or delayed synostosis [65].

## Conclusions

Despite being a rare disease and described anecdotally in the mandible, infantile myofibromatosis is the most common fibrous tumor of infancy. We presented a case of myofibroma of the mandible with very aggressive behavior resistant to all mandibular sparing treatment strategies including chemotherapy and debulking. A hemi-mandibulectomy with initial bridge plating and coverage with submental island flap and delayed reconstruction with fibula free-flap reconstruction was done successfully. This reconstruction strategy is novel and was chosen due to the patient’s very young age at the resection (18 months) and aggressiveness of the disease. To our knowledge, this is the only reported case of fibula free flap mandibular reconstruction in a patient with IM, as well as one of the youngest reported submental island flaps for any pathology. Segmental mandibular deficits are very rare in children and surgical reconstruction is a significant challenge.

## Abbreviations

AP: Anteroposterior
CC: Cephalocaudal
CT: Computerized tomography
IM: Infantile myofibromatosis
MRI: Magnetic resonance imaging
T: Transverse
US: Ultrasound
